# Two Cases of Spontaneous Liver Rupture and Literature Review

**DOI:** 10.1155/1996/24016

**Published:** 1996

**Authors:** P. J. Cozzi, D. L. Morris

**Affiliations:** UNSW Department of Surgery The St. George Hospital Gray Street, Kogarah Sydney NSW 2217 Australia

## Abstract

Spontaneous liver rupture is uncommon, difficult to diagnose and carries a universally high mortality. It has been well documented to occur as a complication of primary or secondary hepatic malignancy. Similarly, there are 28 cases of ruptured haemangiomata described in the world literature. It is also well described in severe pregnancy-induced hypertension and is said to carry a mortality of 18% for patients treated by packing and drainage of the haematoma and 75% for patients treated with liver resection.

Two female patients aged 60 and 61 presented to our accident and emergency department. One had a history of hypertension only and the other a history of a bleeding diathesis from the lupus anticoagulant. Both presented with hypotension and abdominal pain and both were diagnosed by abdominal CT scan. One was treated with hepatic artery ligation and tamponade and the other with liver resection and correction of the coagulopathy. Neither had any evidence of a ruptured haemangioma or tumour at laparotomy or on histological examination, and both are alive and well.

The conclusions to be drawn from this review and our own recent experience is that the treatment of choice for ruptured haemangiomata is liver resection and, for rupture during pregnancy, is tamponade with packs and evacuation of the haematoma. Hepatic arteriography and embolisation, if possible, is a useful adjunct. Correction ofany coagulopathy is essential. We can only speculate that the aetiology in our patients was uncontrolled hypertension in one and coagulopathy in the other.


*HPB Surgery*, 1996, Vol.9, pp.257–260
Reprints available directly from the publisher
Photocopying permitted by license only

© 1996 OPA (Overseas Publishers Association)
Amsterdam B.V. Published in The Netherlands
by Harwood Academic Publishers GMbH
Printed in Malaysia

### CASE REPORT

### Two Cases of Spontaneous Liver Rupture and Literature Review

P. J. COZZI and D. L. MORRIS

UNSW Department of Surgery, The St George Hospital, Kogarah

*(Received 10 April 1993)*

Spontaneous liver rupture is uncommon, difficult to diagnose and carries a universally high mortality. It has been well documented to occur as a complication of primary or secondary hepatic malignancy. Similarly, there are 28 cases of ruptured haemangiomata described in the world literature. It is also well described in severe pregnancy-induced hypertension and is said to carry a mortality of 18% for patients treated by packing and drainage of the haematoma and 75% for patients treated with liver resection.

Two female patients aged 60 and 61 presented to our accident and emergency department. One had a history of hypertension only and the other a history of a bleeding diathesis from the lupus anticoagulant. Both presented with hypotension and abdominal pain and both were diagnosed by abdominal CT scan. One was treated with hepatic artery ligation and tamponade and the other with liver resection and correction of the coagulopathy. Neither had any evidence of a ruptured haemangioma or tumour at laparotomy or on histological examination, and both are alive and well.

The conclusions to be drawn from this review and our own recent experience is that the treatment of choice for ruptured haemangiomata is liver resection and, for rupture during pregnancy, is tamponade with packs and evacuation of the haematoma. Hepatic arteriography and embolisation, if possible, is a useful adjunct. Correction of any coagulopathy is essential. We can only speculate that the aetiology in our patients was uncontrolled hypertension in one and coagulopathy in the other.

KEY WORDS: Spontaneous liver rupture pregnancy-induced hypertension haemangioma

### CASE 1

A 60-year-old woman was transferred from a peripheral hospital to our Accident and Emergency Department with a diagnosis of a ruptured abdominal aortic aneurysm. She had a past history of hypertension only and had recently changed her antihypertensive medication. She gave no history of a bleeding diathesis. She presented with epigastric pain and hypotension. On examination, she had a systolic blood pressure of 70 mm Hg and a pulse rate of 100 b.p.m. The JVP was not raised, there was tenderness to deep palpation in the epigastrium and no palpable abdominal aortic aneurysm. She was resuscitated and then transferred to our unit. In our department, the findings were similar and she was transferred immediately to the operating theatre for an urgent laparotomy. At laparotomy, no aortic aneurysm was found and there was no other finding to account for this patient's presentation. Following improvement in clinical condition, the patient was transferred to the Intensive Care Unit.

The patient continued to be haemodynamically unstable and had a fall in haemoglobin to 65 g/L. A transthoracic and trans-oesophageal echocardiogram was performed and found to be normal. A CT scan of the abdomen was then undertaken (Figure 1). The patient then returned to the operating theatre. At laparotomy, it was found that a subcapsular haematoma of the liver had ruptured into the peritoneum. There was no evidence of a haemangioma. There was massive intraperitoneal bleeding. The hepatic artery was ligated and the liver treated with diathermy and packing. The patient was further resuscitated with blood and blood products to correct transfusion-associated

*Correspondence to*: Professor D. L. Morris, UNSW Department of Surgery, The St. George Hospital, Gray Street, Kogarah, Sydney NSW 2217, Australia, Phone: (61–2) 3502070 Fax: (61–2) 3503997

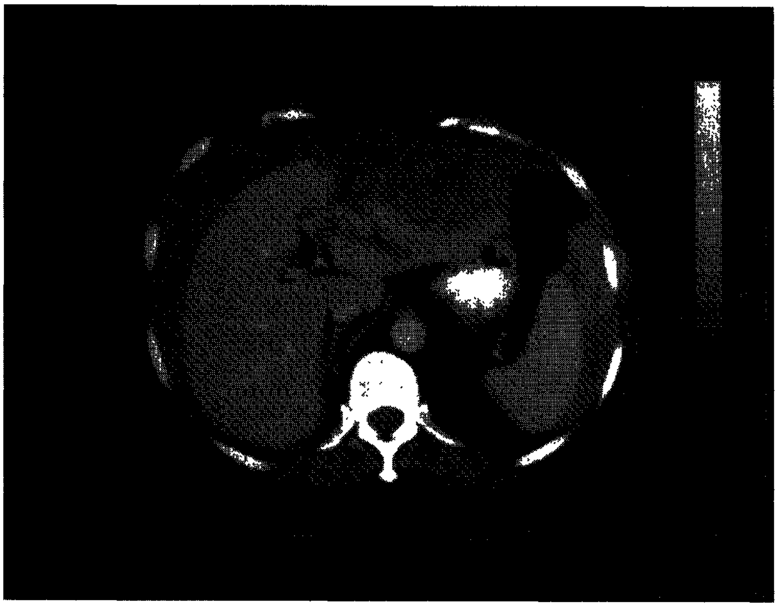

**Figure 1** Abdominal Computerised Tomography (CT) scan of Patient 1.

coagulopathy. Forty-eight hours later, the patient returned to theatre for removal of packs. The post-operative course was complicated by many problems, including gastrointestinal bleeding from both the stomach and the colon, the latter requiring a subtotal colectomy. After a number of radiologically-guided drainages of intra-abdominal collections, an entero-cutaneous fistula occurred. This was treated at laparotomy and the patient now shows no sign of fistula recurrence. Despite a post-operative myocardial infarct and other medical problems, the patient remains well and has had no further bleeding from the liver. There is no evidence of a bleeding diathesis.

### CASE 2

A 61-year old, 100 kg lady presented to our Accident and Emergency Department complaining of a three-day history of epigastric pain. She had a past history of a bleeding diathesis from the lupus anticoagulant which has been previously described[1]. She had had post-operative bleeding after a previous laparotomy, a spontaneous haemoarthrosis of the knee and troublesome bleeding after tooth extraction. She was haemodynamically stable on admission and had tenderness to deep palpation in the epigastrium but was otherwise normal to examination. Her investigations revealed a haemoglobin of 143 g/L, a prothrombin time of 13 (10–13) and an activated partial thromboplastin time (APTT) of 66 (21–35). The patient became haemodynamically unstable with a fall in blood pressure to 80 mm Hg systolic. An urgent abdominal CT scan was performed (Figure 2) prior to urgent laparotomy. At laparotomy, there was massive intra-abdominal bleeding and a large irregular laceration in the left lateral segment. A left lateral segmentectomy was performed using the CUSA and bleeding from the liver edge was controlled with clips, ligatures and by oversewing. After haemostasis was achieved, the abdomen was closed and correction of her coagulopathy was undertaken.

Further haemodynamic instability and a fall in haemoglobin despite resuscitation necessitated a return to the operating theatre, at which time it was found that the bleeding was from multiple sites, including the wound. This was carefully treated with diathermy and packing until haemostasis was again achieved. Plasmapheresis was instituted to remove the anticoagulant from the plasma. The APTT was reduced to 44. The intra-abdominal packs were removed after 48 hours. Laparotomy and packing was required on two further occasions during the subsequent week. A tracheostomy was performed later and this was complicated by bleeding. Bleeding also occurred from the vagina and the rectum. The rectum was found to have a number of superficial ulcers which were treated with diathermy. In all instances, the treatment of the bleeding by tamponade or diathermy was greatly aided by plasmapharesis.

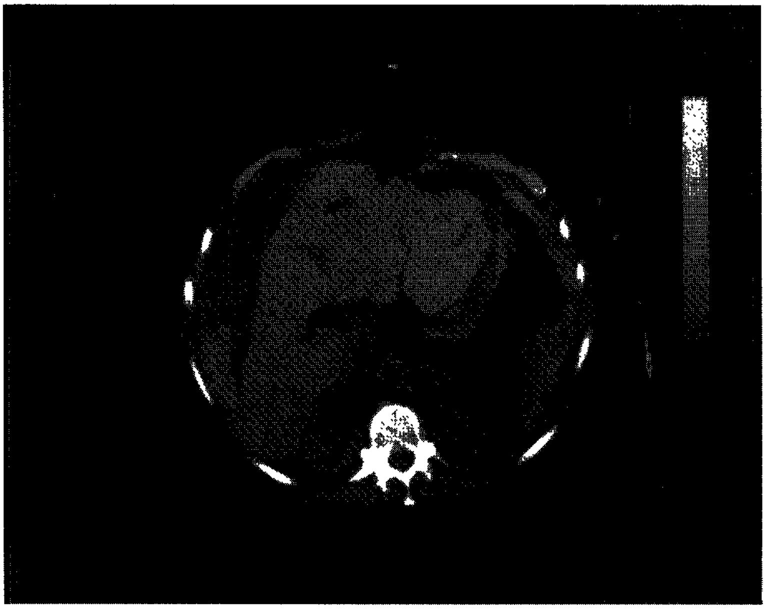

**Figure 2** Abdominal CT Scan of Patient 2.

Following improvement in the patient's clinical condition, she was commenced on cyclophosphamide and prednisolone in an attempt to reduce the production of the anticoagulant. It was further confirmed that the anticoagulant was her only manifestation of systemic lupus erythematosis (SLE) and that she was one of the very few people in the world with the extremely rare condition of the lupus anticoagulant actually causing bleeding. Histological examination of the resected liver showed no evidence of haemangioma or tumour. The patient was discharged after three months in hospital.

### DISCUSSION

First described by Abercrombie in 1844[2], spontaneous rupture of the liver remains a rare condition, characterised by a universally poor prognosis. It is well documented to occur during pregnancy as a complication of pregnancy-induced hypertension and is said to carry a high rate of maternal and infant mortality.[3] It may occur as a complication of hepatocellular carcinoma and has an incidence of rupture of 10.2–14.5% of patients[4,5]. It may also occur in metastatic disease of the liver and is likely to be a terminal event, the incidence of which is probably under-reported. It is also well documented to occur as a complication of hepatic haemangiomata[6]. Less common causes include rupture of focal haemorrhagic necrosis[7] and there is a single case report of a hepatic laceration caused by vomiting[8].

Treatment of this uncommon condition includes hepatic artery ligation[9,10] or embolisation[6,11], hepatic lobectomy[6,12,13], observation[3], evacuation and tamponade with packs[3], application of haemostatic agents[14] and oversewing of the liver[15]. The treatment depends upon an accurate knowledge of the aetiology.

Yamamoto's review[6] of 19 adult cases of spontaneous rupture of liver haemangiomata documents a mortality of 56.3%. In this review, all three patients who had no surgery died, one of three undergoing partial resection died, three of four undergoing formal or extensive lobectomy died, two of five that were sutured died and three of four undergoing tamponade died. He concluded that hepatic artery embolisation should be considered pre-operatively and that arteriography was valuable because it greatly facilitated knowledge of tumour extension.

Smith's review[3] of spontaneous rupture of the liver during pregnancy-induced hypertension in 35 cases found a survival rate of 82% for patients treated with draining and packing, compared to 25% for lobectomy ($p = 0.006$). The diffuse end organ damage that occurs in this condition is reversible after delivery and this explains the success of tamponade followed by confinement.

The aetiology of our cases is obscure. The first case was perhaps due to uncontrolled hypertension and was successfully treated with hepatic artery ligation and tamponade. The second case was related to the coagulopathy but the cause of the initial bleed remains obscure. Perhaps there was a lesion missed on histology. This case was successfully managed by resection of the macroscopically abnormal liver and tamponade. Both patients had aggressive treatment of coagulopathy.

### CONCLUSION

In summary, the treatment of this unusual condition depends on the aetiology. In the diffusely abnormal liver, tamponade would seem the preferred treatment and, in the focally abnormal liver, arteriography and embolisation and/or resection would seem appropriate. The importance of tamponade with packs cannot be overstated. Correction of any pre-existing or iatrogenic coagulopathy is mandatory and should be aggressively pursued.

### REFERENCES

1. Manoharan, A., Gottleib, P.(1984) Bleeding in patients with *lupus anticoagulant* (letter) *Lancet*, **2**: (8395), 171.
2. Abercrombie, J.(1844) Hemmorhage of the liver. *London Medical Gazette*, **34**: 792–4.
3. Smith, L.J., Moise, K.J., Dildy, G.A. and Carpenter, R.J.(1991) Spontaneous rupture of liver during pregnancy: current therapy. *Obstetrics and Gynaecology*, **77**: 171–175.
4. Chearanai, O., Plengvanit, U., Asavanich, C., Damrongsak, D. and Sindhvananda, K. (1983) Spontaneous rupture of primary hepatoma: report of 63 cases with particular reference to the pathogenesis and rationale treatment by hepatic artery ligation. *Cancer*, **51** (8), 1532–1536.
5. Ong, G.B. and Taw, J.L. (1972) Spontaneous rupture of hepatocellular carcinoma. *British Medical Journal*, **4**: 146–149.
6. Yamamoto, T., Karawada, Y., Yano, T., Noguchi, T. and Mizumoto, R. (1991) Spontaneous rupture of haemangioma of the liver: treatment with transcatheter hepatic arterial embolization. *American Journal of Gastroenterology*, **82**: 1645–1659.
7. Takiff, H., Brems, J., Pocktos, P.J. and Elliot, M.L. (1992) Focal hemorrhagic necrosis of the liver. A rare cause of hemoperitoneum. *Digestive Diseases and Sciences*, **37**: 1910–1914.
8. Horvath, K.A. and Glasgow, A.H. (1992) Massive intra-abdominal hemorrhage from a hepatic laceration caused by vomiting. *Archives of Surgery*, **127**: 1361.
9. Gonzalez, G.D., Rubel, H.R., Giep, N.N. and Bottsford, J.E. (1984) Spontaneous hepatic rupture in pregnancy: management with hepatic artery ligation. *Southern Medical Journal*, **77**: 242–245.
10. Mays, E.T., Conti, S. and Fallahzadeh, H. (1979) Hepatic artery ligation. *Surgery*, **86**: 536–543.
11. Loevinger, E.H., Ivutic, I., Lee, W.M. and Anderson, M.C. (1985) Hepatic rupture associated with pregnancy. Treatment with transcatheter embolotherapy. *Obstetrics and Gynaecology*, **65**: 281–284.
12. Utley, J.R. (1971) Spontaneous rupture of the liver during pregnancy. *Surgery, Gynecology and Obstetrics*, **133**: 250–252.
13. Nelson, E.W., Archibald, L. and Albo. D.(1977) Spontaneous hepatic rupture in pregnancy. *American Journal of Surgery* **134**: 817–820.
14. Bis, K.A. and Waxman, B. (1976) Rupture of the liver associated with pregnancy: A review of the literature and report of two cases. *Obstetrical and Gynecological Survey*, **31**: 763–773.
15. Hakin-Elahi (1965) Spontaneous rupture of the liver in pregnancy. Obstetrics and Gynecology, **26**: 435–449.

